# Recreational drug and excessive alcohol use among HIV-infected men who have sex with men in Central Israel

**DOI:** 10.1186/s12889-019-7747-4

**Published:** 2019-10-24

**Authors:** Zohar Mor, Dan Turner, Yuval Livnat, Itzchak Levy

**Affiliations:** 1 Tel Aviv Department of Health, Ministry of Health, Tel Aviv, Israel; 2 School of Health Sciences, Ashkelon Academic College, Ashkelon, Israel; 3 AIDS Treatment Centre, Sourasky medical Centre, Tel Aviv, Israel; 4 Sackler Faculty of Medicine, Tel Aviv University, Tel Aviv, Israel; 5 Israeli AIDS Task Force, Tel Aviv, Israel; 6 Buchman Faculty of Law, Tel Aviv University, Tel Aviv, Israel; 7 AIDS and STD Unit, Sheba Medical Centre, Ramat Gan, Israel

**Keywords:** Alcohol, Chemsex, HIV, Israel, Men who have sex with men, Recreational drug, Sexual risk

## Abstract

**Background:**

HIV-infected men who have sex with men (MSM) who use recreational drugs (RD) or excessive alcohol (EA) may be involved in risky sexual behaviours, including unprotected anal intercourse (UAI). This study describes the prevalence RD/EA-use among HIV-infected MSM, and compares those who used RD/EA with those who did not.

**Methods:**

This cross-sectional study included HIV-infected MSM who were recruited in a convenient sample from two AIDS-treatment centres and events for HIV-infected MSM in Israel in 2016. Participants completed anonymous questionnaires including RD/EA-use and their sexual behaviours. RD/EA-use was defined as consumption of any psychoactive stimulants or dissociative anaesthetics, or an uptake of alcohol until drunkenness before or during sex.

**Results:**

Of all 276 HIV-infected MSM, 202 (73.2%) used RD/EA. Those who used RD/EA were younger, reported earlier sexual debut, had more sexual partners, were more likely to perform UAI with casual partners, more commonly involved in paid sex, used psychiatric medications and more likely to be unsatisfied with their health-status compared to those who did not use RD/EA. HIV-infected MSM who used RD/EA reported a lower CD4-count and higher viral-load than those who did not. In a multivariate analysis, being younger, reported earlier sexual debut and been prescribed psychiatric drugs were associated with RD/EA-use among HIV-infected MSM.

**Conclusions:**

A large proportion of HIV-infected MSM used RD/EA and also engaged in risky sexual behaviours. A subset of HIV-infected MSM can benefit from mental support during their routine treatment at the AIDS treatment centres and should also receive harm reduction intervention by their providers in order to minimize potential risks pertaining to RD/EA-use.

## Background

Men who have sex with men (MSM) are more likely to use substances such as excessive alcohol (EA) consumption, marijuana or recreational drugs (RD) than the general male population [1, 2]. These substances are often coupled with additional risk behaviours, such as unprotected sex anal intercourse (UAI), multiple sex partners and insufficient certainty in serosorting of sexual partners- all conditions that are set for HIV acquisition or transmission [3, 4].

The common consumption of illicit psychoactive RD in order to facilitate sexual pleasure is colloquially termed in Europe “chemsex”, or “party and play” (sometimes abbreviated to PnP) in north America and Australia [5]. It is usually referred to the use of a synthetic amphetamine (methylmethcathinone, mephedrone, 3,4-Methylenedioxymethamphetamine or MDMA), crystallised methamphetamine (a synthetic amphetamine, also termed as ‘crystal-meth’) or the dissociative anaesthetics ketamine and gamma-hydroxybutyrate/gamma-butyrolactone (GHB/GBL). Chemsex sessions can last hours or days and may involve multiple sexual partners who are often identified using smartphone networking applications [6]. These drugs increase the feelings of euphoria, enhance sexual arousal and eroticism, which results in restoring libido and intensifying sexual sensation and pleasure [7].

HIV-infected MSM are more likely to use RD/EA compared with MSM who are HIV-negative or with unknown sero-status [8], including polydrug-use [9]. HIV-infected MSM who are under the influence of substances may facilitate a “cognitive escape”, which is characterized by avoidance from thought related to their HIV infection. Consequently, while those men are under the influence of substances, they may change their behaviour from a constant vigilance of safe sex to more liberal practices and can potentiate further HIV-transmissions in the community [10].

In Israel, 41.5% (*N* = 2052) of all Israeli males citizens men who were diagnosed with HIV between 1981 and 2015 were MSM [11]. Findings from studies performed in the general MSM community in Israel [12] have associated the sexualized use of some RD/EA with UAI, especially methamphetamine and erectile dysfunction drugs. However, no study has yet addressed the patterns of RD/EA use among HIV-infected MSM in Israel. This study compares HIV-infected MSM who used RD/EA with those who did not by their socio-demographic, behavioural and HIV-related factors and their association with UAI.

## Methods

This cross-sectional study included a convenient sample of HIV-infected MSM in Israel who were treated in the two major AIDS treatment centres in central Israel. Study participants were included in this study if they had visited one of the AIDS treatment centres more than once during the last year, in which they were tested for CD4 count and plasma viral load, and also reported that they performed anal sex with another man during the last year.

HIV-infected MSM were approached in two AIDS treatment centres in central Israel 2016, as well as during social events of the Israel AIDS Task Force targeting HIV-infected MSM. Men attending the clinic and events were requested to complete self-administered anonymous questionnaires. The questionnaires were adaptive, and participants were asked different questions, depending on their sexual behaviours and type of partners. Participants were asked to complete their demographic details, as well as their sexual behaviours, condoms use or UAI, utilization of medical services, RD/EA-use and their last CD4 counts and viral loads (Additional file 1).

RD/EA-use was defined as consumption of any illicit drug, such as cannabis/marijuana, MDMA/GHB/GBL/ketamine/crystal meth or any psychoactive stimulant or an uptake of alcohol until drunkenness [13, 14] immediately before or during sexual intercourse in the last six month. Type of partnerships were classified as reported by study participants: a steady partner was defined as committed relationships; a sex buddy- if they had non-committed regular sexual relationships; and a casual partner- if it was a ‘one night stand’.

### Statistical analysis

Dependent variable in this study was a self-report of RD/EA-use. Characteristics of HIV-infected MSM who used RD/EA were compared with those who did not use. Univariate analysis was performed by the chi-square test for categorical variables and by the Student *t*-test for continuous variables in case they were normally distributed or the Mann-Whitney test for variables which were not distributed normally. *P* values lower than 5% were regarded as statistically significant. Variables which were statistically significant in the univariate analysis were included in the multivariate analysis performed by a logistic regression model to identify attributes associated with RD/EA-use, after excluding collinearity between the independent variables, generating odds ratios (OR) and 95% confidence intervals (95% CI).

## Results

This study included 276 HIV-infected MSM who performed anal sex in the last year, with a mean age of 39.1 ± 9.7 years, mostly were Israeli-born Jewish men (Table 1). The majority defined themselves as gay men (95.8%), and have disclosed their sexual orientation (97.3%) and also their HIV infection (75.9%) to significant persons.

**Table 1:** Socio demographic characteristics of the study sample

Question (number responded)	N (%)
Age in years, mean (±SD)	39.1 ± 9.7
Israeli born (*N* = 270)	243 (90.0)
Less than the average income (*N* = 270)	169 (62.6)
University education (*N* = 270)	151 (55.9)
Defined as a gay man (*N* = 167)	164 (98.2)
Jewish (*N* = 208)	200 (96.1)
HIV “outness” (*N* = 274)	198 (72.2)
Gay “outness” (*N* = 164)	109 (66.4)

SD- Standard deviation

Of all study participants, 202 (73.2%) used RD/EA during or before sex within the last six months, while 74 (26.8%) have not. Those who used RD/EA were younger than those who did not, reported earlier sexual debut, had more casual partners, were more likely to perform UAI with sero-discordant partners, and more commonly involved in paid sex (Table 2). HIV infected MSM who used RD/EA reported that the issue of HIV is more commonly raised during or before sex with their encounters compared to HIV-infected MSM who did not use substances. No statistical differences were found in the rate of self-reported sexually transmitted infection (STI) between the two groups. Those who used RD/EA were more commonly prescribed psychiatric medications by their providers and were more likely to be unsatisfied with their health status compared to those who did not use RD/EA. Men who used RD/EA reported lower CD4 counts and viral loads.

**Table 2:** Comparison of demographic and behavioural characteristics in the last six months between HIV-infected MSM who used recreational drugs or excessive alcohol and those who did not use

	Characteristic	Recreational drugs or excessive alcohol *N* = 202	No drugs or alcohol *N* = 74	*P*
Demographics	Age	37.8 ± 9.3	43.2 ± 10.9	< 0.001
	Israeli born	180 (90.0)	63 (85.1)	0.2
	Less than the average income*	125 (62.2)	44 (59.9)	0.4
	University education*	109 (54.2)	42 (56.8)	0.4
	Defined as a gay man	125 (98.4)	39 (92.9)	0.09
	Jewish	145 (92.9)	55 (96.5)	0.6
	HIV “outness”	148 (73.6)	50 (79.5)	0.2
	Gay “outness”	81 (96.4)	28 (96.6)	0.7
Sexual behaviour	Age first sexual debut (years)	16.5 ± 3.9	18.9 ± 5.5	< 0.001
	Steady partner	78 (38.6)	25 (33.8)	0.3
	Partner is HIV infected or his status was unknown	28 (36.5)	5 (20.8)	0.2
	Performs UAI with sero-discordant steady partner	19 (9.4)	7 (9.5)	0.6
	Performs UAI with steady partner	42 (57.5)	11 (45.8)	0.2
	Sex buddy	71 (78.0)	19 (82.6)	0.4
	Sex buddy is HIV-infected or his status was unknown	10 (5.0)	1 (3.2)	N/A
	Performs UAI with sero-discordant sex buddy	10 (5.0)	0 (0)	N/A
	Sex with casual partner/s	165 (81.7)	53 (71.6)	0.05
	> 11 casual partners	79 (45.7)	16 (28.6)	0.02
	Sex with casual partners who was HIV-infected or unknown	163 (96.4)	52 (94.5)	0.4
	Performs UAI with sero-discordant casual partner	75 (59.5)	16 (40.0)	0.04
	Been paid for sex	44 (22.1)	6 (8.1)	0.005
Talking about HIV	HIV issue is being raised in most times	116 (57.4)	29 (39.9)	0.002
	I am the one who raise the issue of HIV	72 (43.4)	17 (37.0)	0.2
Venue of meeting the partners	Applications	167 (87.4)	55 (80.9)	0.1
	Facebook	21 (11.6)	8 (12.3)	0.5
	Sauna	27 (15.0)	8 (12.3)	0.4
	Club	44 (23.9)	6 (9.1)	0.06
Sexually transmitted infections	Penile condyloma	14 (6.9)	4 (5.4)	N/A
	Anal condyloma	48 (23.8)	14 (18.9)	0.4
	Neisseria gonorrhoea	61 (30.2)	17 (23.0)	0.1
	Syphilis	51 (25.2)	17 (23.0)	0.7
	Hepatitis C	9 (4.5)	1 (1.4)	N/A
	Hepatitis B	7 (3.5)	2 (2.7)	N/A
Utilization of health services	Psychologist	78 (39.6)	20 (28.6)	0.1
	psychiatric medications	67 (33.2)	14 (19.7)	0.01
	Satisfied with their health status	169 (86.7)	66 (95.7)	0.03
	Years of HIV infection	5.2 ± 4	6.6 ± 3	0.06
	Anti-retroviral treatment	143 (94.1)	56 (93.3)	0.5
	Treated with anti-retroviral medications > 2 years	95 (66.4)	41 (73.2)	0.2
	Complete treatment adherence	97 (69.8)	38 (67.9)	0.9
	Complete check-up adherence	19 (97.8)	49 (84.5)	0.4
	Satisfied from the AIDS treatment centre	135 (90.0)	51 (88.0)	0.9
	CD4	626 ± 237	716 ± 225	0.02
	Viral load	1624 ± 556	988 ± 353	0.03

MSM- Men who have sex with men

UAI- Unprotected anal intercourse

*Age adjusted

In the multivariate analysis, younger age, early sexual debut and been prescribed psychiatric medications predicted the use of RD/EA use among HIV-infected MSM (Table 3).

**Table 3:** Multivariable analysis predicting the use of used recreational drugs or excessive alcohol among HIV-infected MSM

Characteristic	Odds ratio (95% confidence intervals)
Age	1.2 (1.1–1.3)^a^
Age first sexual debut (years)	1.2 (1.1–1.4)^a^
Been paid for sex	2.5 (0.5–3.8)
HIV issue is being raised in most times	1.3 (1.2–2.7)
Psychiatric medications	2.1 (1.3–4.3)
Satisfied with their status	2.4 (0.7–4.4)
CD4 count	1.1 (0.9–1.3)
Viral load	1.0 (0.8–1.1)

MSM- Men who have sex with men

UAI- Unprotected anal intercourse

^a^One year interval

## Discussion

The majority (73.2%) of all HIV-infected MSM in our study used RD/EA in the last six months. Those who used RD/EA were more commonly younger, reported earlier sexual debut and were prescribed psychiatric medications compared with HIV-infected MSM who did not use RD/EA.

GHB/GBL is a nervous system depressant [15], while mephedrone and crystal methamphetamine are stimulants. All three can trigger feelings of euphoria, and also facilitate intense feelings of sexual arousal among many users. This is particularly the case for crystal methamphetamine [16]. Although chemsex is a relatively new phenomenon, it has gradually become normalized within the social and sexual contexts among fragments of the gay community [17], especially in HIV-infected MSM [18]. Several theories have been endorsed to explain the increased use of RD/EA among HIV-infected MSM. These include the attempt to overcome lack of confidence or to ameliorate negative feeling such as low self-esteem, internalized homophobia or stigma associated with being HIV-infected [5]. Substances-use may also mitigate feelings of depression as a result of the HIV-infection [18, 19], as found in our study, where HIV-infected MSM who used RD/EA were more likely to be prescribed psychiatric medications than those who did not use substances. Additionally, RD/EA may reduce body image anxiety, such as lipodystrophy which was caused by older generations of antiretroviral treatment. The substances also facilitate social and sexual connection by moderating fear of being rejected by sexual encounter as for the HIV-infection [16].

HIV-infected MSM who used RD/EA in our study were engaged in UAI more commonly than those who did not, as they might misjudged the sexual risks while being under the influence the substances. HIV-infected MSM in our study also performed UAI with sero-discordant sexual partners, in line with other publications [1]. Chemsex substances increase sexual arousal and confidence, and lead to sexual permissiveness and adventurism. This may result in a high turnover of partners and diverse sexual practices, and play a role in transmitting/acquiring HIV/STI [19], including hepatitis C virus [20].

Younger HIV-infected participants in our study were more likely to use RD/EA, parallel with other publication [21]. Young MSM are more likely to frequent gay-venues, where they are exposed to RD/EA-use behaviour, which has been the norm in certain premises, such as clubs or saunas. They also generally tend to take greater risks than older men, including engaging in UAI [22, 23]. Contrarily, older men may be more concerned with possible sexual risks, and have developed harm reduction strategies, such as sero-sorting to avoid sero-discordant sexual partnering. It is also possible that older HIV-infected MSM were in stable partnerships, thus less exposed to social drug use [24].

Interestingly, HIV-infected MSM participants were followed-up in two major specialized AIDS treatment centres, yet they reported UAI with sero-discordant sex partners although they were aware of their HIV-infection. HIV-infected MSM in our study who used RD/EA had lower CD4 counts and viral loads compared with those who did not use substances. This sexual behaviour reflects the increasing evidence-base information that the risk of HIV transmission from infected person who has achieved an undetectable viral load for at least 6 months is negligible [24].

The high prevalence of RD/EA use among HIV-infected MSM highlights the need to adopt innovative strategies in the AIDS treatment centres, which include drug and alcohol history taking for each patient and address the context of consumption. Exhaustive use of ‘crystal meth’ may cause acute mental distress, and overdosing from GHB/GBL may result in unconsciousness (colloquially known as a ‘G-hole’) and can lead to respiratory depression or choking [25]. Psychiatric counselling or treatment should be offered in cases of uncontrolled use. Additionally, practitioners at the AIDS centres should be able to suggest strategies for ‘safer use’ of RD/EA to avoid or reduce health complications. The high prevalence of UAI among HIV-infected MSM which is related to RD/EA use once again stress the importance of adherence to antiretroviral treatment resulting in viral undetectability, as a method of HIV prevention. Practitioners at the AIDS treatment centres should also be aware of possible chemical interactions between RD/EA and some of the antiretroviral medications, such as non-nucleoside reverse transcriptase inhibitors, which may decrease drug bioavailability and reduce pharmacokinetic characteristics of the active compound [26]*.*

Other harm reduction interventions to reduce harm of RD/EA should be employed in the community to support to HIV-infected MSM. Factual, honest and non-judgmental information about safe use of these substances can be distributed by online and mobile technologies that render gay bars and clubs, which become less central to MSM socialization culture [27]. Another alternative has been successfully implemented in specialist clinics in London, England, where gay men can access harm reduction interventions that were culturally sensitive [25]. Gay-oriented services potentially provide the preferred setting for treatment, rather than traditional drugs services. Traditional drug services have acquired wide experience in managing opiate use among predominantly heterosexual populations, and might not be appropriately skilled to address the needs of gay active men and their specific drug use behaviours [25]. Nonetheless, sexual and RD/EA use habits are often difficult to change, and health practitioner should acknowledge that some HIV-infected MSM enjoy this kind of liberation and engage in certain sexual practices despite of their HIV-infection.

Although it is the first study describing RD/EA use behaviours among HIV-infected MSM in Israel, it may be subjected to several limitations. First, sexual behaviours and RD/EA use may be underreported or associated with a recall bias. In order to minimize the information bias, the questionnaires were completed by the participants anonymously and pertained for behaviours in the last six months. Nonetheless, these biases, if occurred, could have led to underestimation of prevalence, which is conservative in nature. Second, the cross-sectional design of this study may not allow for the establishment of causal links between RD/EA-use and UAI. Yet, the association between RD/EA-use and higher-risk sexual behaviours has been also documented in other publications. Third, this study is limited by the relatively small sample size recruited through convenience sampling methods, and therefore a caution should be made in generalization to all MSM with HIV.

## Conclusion

A large proportion of HIV-infected MSM in our study used RD/EA. Those who used substances were more commonly younger, engaged in risky sexual behaviour and used psychiatric medication compared with HIV-infected MSM who did not use. Gay-oriented interventions should be employed to increase the awareness of HIV-infected MSM about the potential risks pertained to RD/EA-use, and to train these men to employ precautionary measures to prevent self-injury and minimize HIV/STI spread.

## Supplementary information

**Additional file 1.** Study questionnaire.

## Data Availability

The datasets used and/or analysed during the current study are available from the corresponding author on reasonable request.

## Abbreviations

AE: Excessive alcohol
GHB/GBL: Gamma-hydroxybutyrate/gamma-butyrolactone
HIV: Human immunodeficiency virus
MDMA: 3,4-Methylenedioxymethamphetamine
MSM: Men who have sex with men
RD: Recreational drugs
UAI: Unprotected anal intercourse
